# Fluctuating temperatures have a surprising effect on disease transmission

**DOI:** 10.1371/journal.pbio.3002288

**Published:** 2023-09-08

**Authors:** Marta S. Shocket

**Affiliations:** Lancaster Environment Centre, Lancaster University, Lancaster, United Kingdom

## Abstract

Theory predicts that temperature fluctuations should reduce performance near an organism’s thermal optimum. This Primer explores a new study in PLOS Biology showing that fluctuations increased parasite transmission instead, highlighting questions about how climate change will impact infectious diseases.

## Teaser

Theory predicts that temperature fluctuations should reduce performance near an organism’s thermal optimum. A new study in *PLOS Biology* found fluctuations increased parasite transmission instead, highlighting questions about how climate change will impact infectious diseases.

How will shifting temperatures due to climate change impact transmission of infectious diseases? This question sits at the intersection of 2 of the most critical and active areas of ecological research, with important applications for public health, conservation, and agriculture. Answering the question remains an ongoing challenge, and a surprising result from a new paper in *PLOS Biology* by Krichel and colleagues [1] testing the impact of temperature fluctuations on pathogen transmission has added another wrinkle to the current framework.

**Fig 1:**
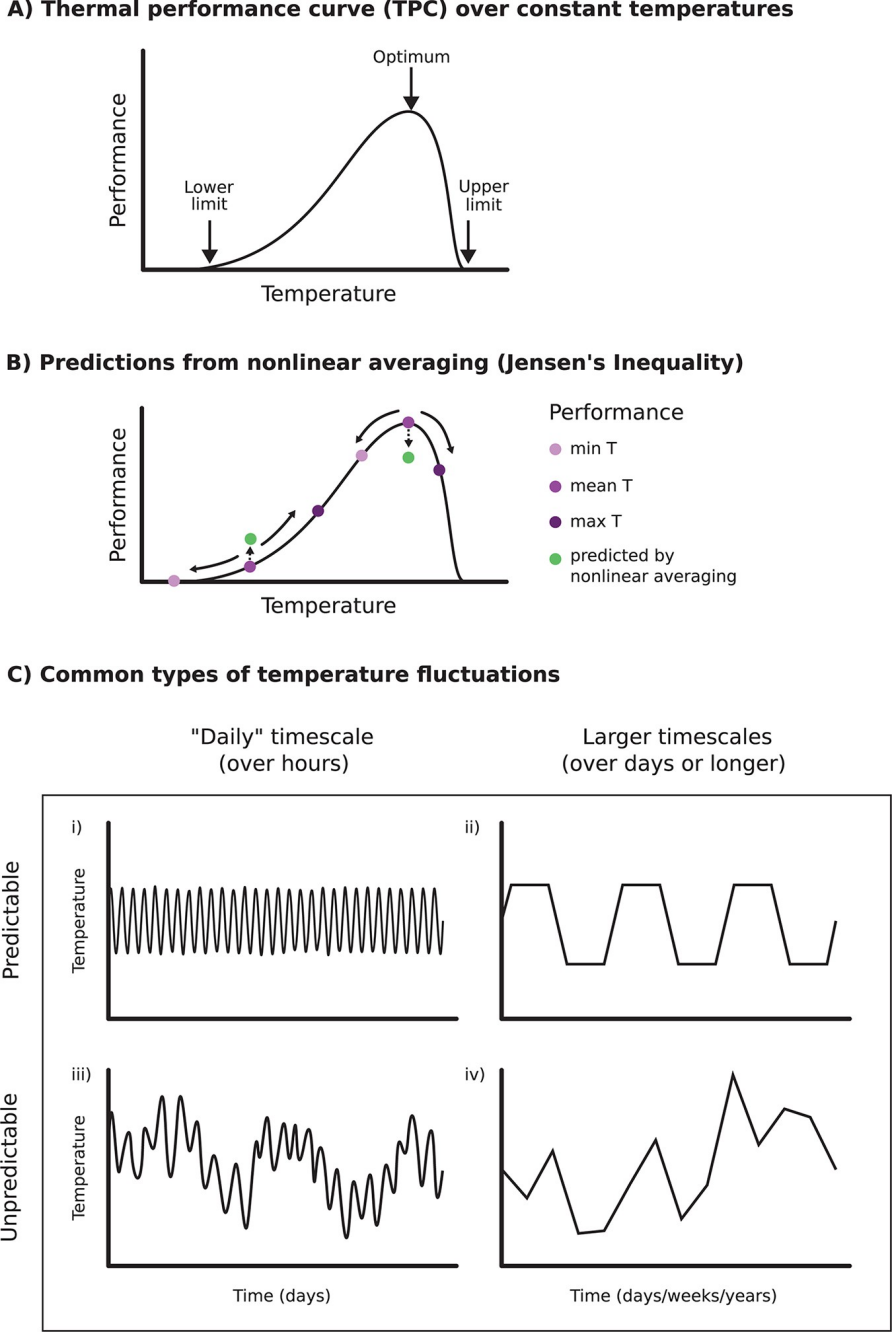
Predicting the impact of fluctuating temperatures on organismal performance and ecological outcomes. (A) A stereotypical TPC fit based on measurements in constant temperature environments. As temperature increases, performance initially also increases from a lower limit, then reaches its maximum value at the optimal temperature, and finally decreases to the upper limit. In some cases, this pattern also scales up to species interactions like disease transmission, often predicted by a mathematical model parameterized with organismal traits. (B) Nonlinear averaging predicts performance in fluctuating environments by assuming that performance (or ecological outcome) follows the constant temperature TPC and performance changes instantaneously with the environment. Fluctuations reduce predicted performance if the curve is decelerating or concave down (e.g., near the optimum), because the organism spends little time at the ideal temperature. Fluctuations increase predicted performance if the curve is accelerating or concave up. (C) The impact of temperature fluctuations and the ability of nonlinear averaging to accurately predict performance or ecological outcomes may depend on the timescale or predictability of the fluctuations. Thermal fluctuations can occur over “daily” (left column) or longer (right column) timescales, and fluctuations at both timescales may be either predictable (top row) or unpredictable (bottom row). Most studies focus on just 2 combinations of timescale and predictability: (i) predictable “daily” fluctuations and (iv) unpredictable fluctuations over larger timescales. (Note: fluctuations between the daily maximum and minimum temperatures [e.g., day vs. night; left column] are commonly called “daily” or “diurnal” temperature variation, although temperature is changing at the hourly timescale).

Thermal biologists and ecologists have known for decades that ectothermic organisms typically respond to increasing temperature in a predictable, unimodal way: their performance initially increases, reaches its maximum value, and then decreases as temperature continues to rise (Fig 1A) [2]. However, it has proven much more challenging to accurately predict the impact of temperature on species interactions like parasitism, even when we have a good understanding of how the more complex ecological outcome depends on simpler organism-level traits. Although the general shape of thermal performance curves (TPCs) for organismal traits is usually consistent (Fig 1A), different species can vary substantially in the steepness of their response and the optimal temperature where they perform best [3,4]. Thus, the TPCs of 2 or more interacting species can theoretically combine in multiple different ways [3,5]. For a host and its parasite, each organism may perform better relative to the other over different sections of a temperature gradient [3,5], or there may be multiple organism-level traits that contribute to transmission and respond differently to temperature [4,6]. Nonetheless, it seems that for many infectious diseases the thermal response of transmission follows a unimodal shape that is similar to the stereotypical TPC for organismal traits (Fig 1A), peaking at an intermediate “Goldilocks” temperature that is neither too hot nor too cold [4,6,7].

Fluctuations around a mean temperature—an inherent part of most natural environments—add yet another layer of complexity to predicting organismal performance and species interactions across temperature gradients. It is generally infeasible to conduct experiments with treatments at enough relevant combinations of mean temperature and fluctuation size. Thus, ideally there would be a modeling approach that could accurately predict organismal performance and ecological outcomes in fluctuating environments based on performance observed in constant temperatures. Nonlinear averaging is a commonly used method (e.g., [8]) that assumes: (1) performance follows the same TPC measured under constant temperatures; and (2) performance changes instantaneously with the environmental temperature. One important feature of predictions made using nonlinear averaging is that the performance in fluctuating conditions will differ systematically from performance in a constant environment with the same mean temperature, as described by a mathematical property called Jensen’s inequality [8,9]. Fluctuations should reduce performance if the curve is decelerating or concave-side down (e.g., near the optimum of a unimodal TPC), because the organism spends little time at the ideal temperature, even if the mean temperature is near the optimum; conversely, fluctuations should increase performance if the curve is accelerating or concave-side up, as can occur in other sections of the curve (Fig 1B). While nonlinear averaging seems to perform well in some cases [8], it has not been widely validated, particularly for thermal responses for transmission of infectious diseases.

In their recent paper, Krichel and colleagues [1] investigated the impact of temperature fluctuations on pathogen transmission in experimental mesocosms and generated a surprising result. Transmission of the intracellular parasite (microsporidian *Ordospora colligata* that infects freshwater zooplankton *Daphnia magna*) responds unimodally to constant temperatures [7]. Thus, nonlinear averaging predicts that fluctuations around the optimal temperature should decrease transmission compared to a constant environment with the same mean (Fig 1B). However, their experiment found the opposite effect instead: thermal fluctuations increased the prevalence of parasites, as well as the intensity of infection per host [1]. Few other studies have directly tested the effect of fluctuating temperature on pathogen transmission. Generally, results from these studies have qualitatively matched the theoretical predictions, with fluctuations decreasing prevalence (e.g., [10,11]), although 1 study conducted using the same host–parasite system as Krichel and colleagues [1] found little change in prevalence near the optimum [12]. Observational studies using human epidemiological data have also found that larger daily temperature fluctuations lower disease transmission (e.g., [13]).

So what could possibly be driving the disparity between theoretical predictions and empirical results in this case? One potential answer is acclimation, which allows organisms to maintain high levels of performance in variable environments. The temperature variability hypothesis suggests that parasites can gain an advantage in fluctuating thermal environments because they are smaller than their hosts and therefore can acclimate faster to changing temperatures [14]. This hypothesis was developed from work in an amphibian-chytrid fungus disease system where a temperature shift also increased infection intensity per host but did not affect prevalence [14]. Another set of possibilities is that the predictability of temperature variation or the timescale of fluctuations relative to the biological processes underlying transmission is key (Fig 1C). The prior study that investigated the impact of fluctuating temperatures on transmission in the *Ordospora-Daphnia* disease system used consistent “daily” fluctuations that changed the temperature each hour (Fig 1C, subpanel i) [12] while Krichel and colleagues used fluctuations that changed the temperature each day to produce a random walk over a longer timescale (Fig 1C, subpanel iv) [1].

Overall, the study by Krichel and colleagues [1] demonstrates the utility and importance of ecological research that tests theory by combining predictive modeling and manipulative experiments. It also highlights just how many open questions remain regarding how climate change will impact future transmission of infectious diseases and how fluctuating temperatures affect species interactions. Are certain types of organismal traits or species interactions more likely to match the predictions made by nonlinear averaging? Is nonlinear averaging more likely to work for certain types of thermal variation (e.g., predictable versus unpredictable fluctuations or fluctuations at specific timescales; Fig 1C)? Does it make sense to perform nonlinear averaging directly on the thermal response for complex ecological outcomes like infection prevalence, or should we do it on the TPCs for the underlying organismal traits that are used to parameterize the population model? Can we develop a better model for predicting performance in fluctuating temperatures, perhaps one that incorporates a more mechanistic understanding of acclimation, heat stress, or other aspects of within-organism biology? Do we need cross-scale models that link within-host dynamics to among-host transmission? More research across a variety of host–parasite systems, thermal regimes, and biological scales is necessary to find answers to these questions and develop a more robust predictive framework.
